# Pediatric Acute Poisoning: The Bipolar Evolution of the Poisoning Spectrum from 2019 to 2024 in Southwest China

**DOI:** 10.3390/jcm15135309

**Published:** 2026-07-07

**Authors:** Shunli Liu, Yan Wang, Lan Huang

**Affiliations:** 1 Department of Emergency, West China Second University Hospital, Sichuan University, Chengdu 610041, China; liushunli@scu.edu.cn (S.L.); wy20077019@scu.edu.cn (Y.W.); 2 Key Laboratory of Birth Defects and Related Diseases of Women and Children, Sichuan University, Ministry of Education, Chengdu 610041, China; 3 Department of Pediatrics, West China Second University Hospital, Sichuan University, Chengdu 610041, China

**Keywords:** pediatric poisoning, intentional self-harm, psychotropic drugs, epidemiological characteristics

## Abstract

**Objective**: To analyze the evolving epidemiological characteristics of pediatric poisoning from 2019 to 2024 in Southwest China, explore the changing patterns of pediatric poisoning in the post-pandemic era, and provide a reference for poisoning prevention and the development of effective prevention and treatment strategies. **Methods**: This study included 3923 cases of pediatric poisoning treated at the Emergency Department of West China Second University Hospital, Sichuan University. Clinical data such as gender, age, poisonous substance, and cause of poisoning were described. The chi-square trend test was used to analyze annual changes, and multivariate Poisson regression was employed to identify risk factors for hospitalization and for intentional poisoning in children. **Results**: The total number of cases increased significantly from 544 in 2019 to 1006 in 2024. A marked “polarization” pattern was observed: among children aged 1–3 years, unintentional ingestion of household chemicals predominated (*n* = 2332), whereas among adolescents aged 12–14 years, intentional self-poisoning cases surged by 580%, with the toxic agents shifting mainly to psychotropic prescription drugs. From 2019 to 2024, the proportions of intentional poisoning, psychotropic drug poisoning, psychiatric comorbidity, and delayed presentation all increased significantly. Poisson regression indicated that the post-pandemic period, psychiatric comorbidity, and exposure to psychotropic drugs were risk factors for intentional poisoning. **Conclusions**: Following the COVID-19 pandemic, mental health problems among adolescents have become increasingly prominent, and pediatric poisoning has exhibited a bipolarization pattern. Clinical prevention and control strategies should shift from simple emergency treatment to early intervention, psychological screening, and comprehensive prevention, so as to reduce health damage to children and the societal disease burden.

## 1. Introduction

Poisoning remains one of the leading causes of accidental injury and premature death among children and adolescents worldwide [1]. The World Health Organization (WHO) notes that poisoning not only causes severe physical harm and psychological trauma to victims but also imposes a substantial medical and economic burden on families and society [2]. Although the case fatality rate of poisoning has declined over the past decades due to strengthened legislation (e.g., safety packaging) and health education, poisoning continues to be a common presentation in pediatric emergency departments, particularly in many low- and middle-income countries [3].

Regarding epidemiological characteristics, the triggers of poisoning differ significantly between children and adolescents. Poisoning in children under five years of age is generally considered “unintentional” and is closely related to their stage of physiological development. At this age, children have a strong urge to explore but lack the ability to recognize danger, often leading to accidental ingestion of improperly stored household chemicals (e.g., cleaning products), medications, or toxic plants via hand-to-mouth contact [4]. In contrast, poisoning in adolescents is more complex, shifting from “accidental” to “intentional”. Studies have shown that among poisoning cases in adolescents over 12 years of age, a large proportion are associated with intentional self-harm, suicidal intent, and substance/medication abuse [5]. Psychological stress, school conflicts, family tensions, and substance dependence are major risk factors for intentional poisoning in adolescents [6].

The types of toxic agents also vary by geographical environment, socioeconomic level, and age distribution. In developing countries, poisoning with pesticides (e.g., organophosphates) and fuels (e.g., kerosene) remains relatively common, whereas in urbanized areas, prescription drugs (e.g., antipyretics-analgesics, psychotropic medications) and household chemicals are the main sources of poisoning [3,7]. In recent years, improved quality of life has increased awareness of mental health disorders in children, but at the same time, the risk of adolescent exposure to psychotropic drugs has also risen. The outbreak of the 2019 novel coronavirus (COVID-19) pandemic—with multiple factors such as home isolation, a shift in healthcare resources, and increased psychosocial stress—has shaped the pattern of adolescent poisoning, making it a critical public health concern for children that demands urgent attention [8,9].

In-depth investigation of the epidemiological characteristics and risk factors of pediatric poisoning is of great significance for developing targeted prevention strategies, optimizing clinical diagnostic and treatment pathways, and improving public health policies. This study focuses on the evolving patterns of pediatric poisoning, with an emphasis on analyzing the dynamic changes between accidental and intentional poisoning. Considering the background of social change and the impact of the COVID-19 pandemic, we integrate existing evidence to clarify the trends in poisoning patterns across different age groups, identify key influencing factors, and highlight current challenges in prevention and control. Our goal is to provide a scientific basis for developing targeted pediatric poisoning prevention strategies, optimizing family supervision guidance, improving psychosocial support systems, and refining poison control measures, thereby helping to reduce the morbidity and mortality of pediatric poisoning and safeguard children’s physical and mental health.

## 2. Materials and Methods

This was a retrospective cohort study. The study population comprised pediatric patients with acute poisoning (aged ≤14 years) who presented to the Emergency Department of West China Second University Hospital, Sichuan University, between 1 January 2019 and 31 December 2024. Cases were identified through the hospital’s case screening system. Patient information was extracted from the hospital’s electronic medical record system. Two trained researchers independently entered the data into an Excel database, and after data entry, cross-verification was performed to ensure accuracy. For cases with discrepancies, a third researcher reviewed the original medical records and made a final determination, resulting in a complete dataset. Data cleaning involved manual verification of potential outliers against the original medical records. Missing data were neither imputed nor replaced by assumptions. For critical variables with unresolved missing data (i.e., where verification against the original records was not possible or the data remained unavailable), the corresponding cases were excluded from the relevant analyses, and the reasons for exclusion were clearly recorded.

Inclusion criteria: (1) age ≤ 14 years; (2) met the diagnostic criteria for acute poisoning; (3) complete medical records (including key information such as age, sex, time of poisoning, type of toxic agent, poisoning intent, time of presentation, and clinical outcome). Exclusion criteria: (1) chronic or subacute poisoning; (2) cases of adverse drug reactions (e.g., vincristine, methotrexate); (3) endogenous acidosis (including diabetic ketoacidosis, renal tubular acidosis, respiratory acidosis, metabolic acidosis, etc.) and bacterial food poisoning; (4) simple ingestion of non-toxic foreign bodies; (5) medical records lacking core clinical information (e.g., unclear type of toxicant or undetermined poisoning intent); (6) repeated hospital visits and duplicate medical records.

Based on China’s COVID-19 policies and prior literature, we divided the observation period into three stages: pre-pandemic (before December 2019), strict control period (January 2020–December 2022), and post-adjustment period (January 2023 onwards). The middle stage featured nationwide rigorous containment, whereas the later stage saw full optimization of epidemic policies [10,11,12]. The age stratification employed in the present study was as follows: infants (<1 year), toddlers (1–3 years), preschoolers (4–6 years), school-aged children (7–11 years), and adolescents (12–14 years).

Clinical data of children with acute poisoning were statistically described, including general characteristics (age, sex, underlying diseases), place of poisoning, toxic substances, and cause of poisoning. Statistical analyses were performed using Stata 18.0 software (StataCorp LLC, College Station, TX, USA), and Sankey diagrams were drawn using R software version 4.5.3 (R Foundation for Statistical Computing, Vienna, Austria). Descriptive statistics were applied to characterize baseline patient characteristics, including age, sex, comorbidities, toxic agent category, and diagnostic and treatment profiles; categorical variables were summarized as counts (*n*) and proportions (%). For trend analysis, the Cochran–Armitage trend test was performed to assess temporal trends in the proportions of intentional poisoning, psychotropic drug poisoning, and delayed presentation from 2019 to 2024, with results reported as z statistics. We adopted Zou (2004) [13] modified multivariable Poisson regression to explore independent risk factors for intentional poisoning in adolescents aged 12–14 years. The outcome was coded as 0 (unintentional poisoning) or 1 (intentional poisoning). Because intentional poisoning occurred frequently (62.3%) in this group, standard logistic regression would overestimate risk. We thus used Poisson regression with robust standard errors to calculate relative risks (RRs) and 95% confidence intervals (CIs). Clinically meaningful or statistically significant variables were included in the final model. The Wald χ^2^ test assessed model fit. All tests were two-tailed, with *p* < 0.05 indicating statistical significance [13].

## 3. Results

### 3.1. General Characteristics of Children with Acute Poisoning

**Figure 1:**
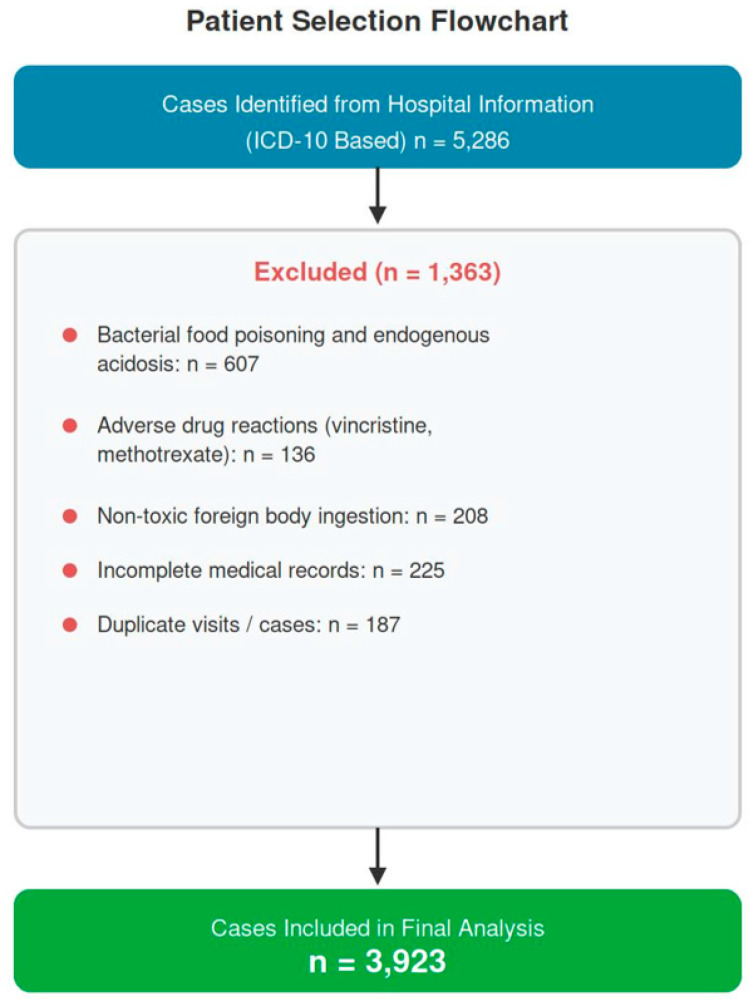
Flow chart of case screening.

This study utilized the hospital information system (HIS) based on ICD-10 coding to preliminarily collect a total of 5286 clinical cases across various diagnostic categories. Sequential screening was performed according to predefined inclusion and exclusion criteria: 607 cases of bacterial food poisoning and endogenous acidosis (including ketoacidosis, renal tubular acidosis, respiratory acidosis, metabolic acidosis, etc.) were excluded, along with 136 cases of conventional adverse drug reactions induced by antineoplastic agents (vincristine, methotrexate), 208 cases of simple ingestion of non-toxic foreign bodies, 225 cases with incomplete medical records, and 187 cases of repeated visits or duplicate patient records. A total of 3923 pediatric poisoning cases were included in this study (Figure 1).

Regarding age distribution, the largest group was children aged 1–3 years (*n* = 2332, 59.4%), followed by those aged 12–14 years (*n* = 531, 13.5%), 4–6 years (*n* = 441, 11.2%), and 7–11 years (*n* = 339, 8.6%); the smallest group was infants <1 year (*n* = 280, 7.1%). In the 12–14 years age group, the proportion of females was significantly higher, accounting for 73.3% of that subgroup. In terms of time to presentation, the 1–3 years age group presented most promptly, with 64.3% of children attending within 4 h. The 12–14 years age group showed the most pronounced delay in presentation, with 63.1% presenting after more than 4 h. The proportion of children with pre-existing psychiatric or psychological disorders was 45.8% in the 12–14 years age group, while the prevalence of psychiatric comorbidity in the other age groups was extremely low. All children under 6 years of age had unintentional poisoning, whereas in the 12–14 years age group, 331 cases (62.3%) were intentional poisoning. The majority of pediatric poisoning cases occurred in the home.

**Figure 2:**
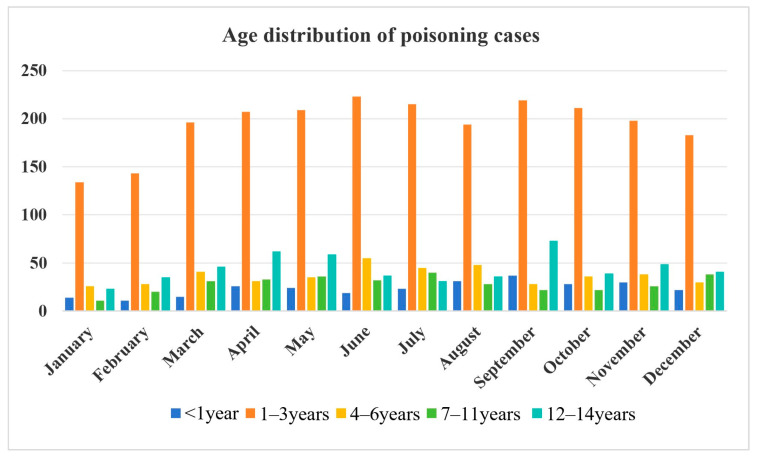
Monthly distribution of poisoning cases across different age groups.

Poisoning in children under 12 years of age was predominantly due to conventional over-the-counter drugs and household chemicals. In contrast, poisoning in children aged 12–14 years was predominantly due to psychotropic drugs. In the vast majority of age groups, poisoning involved a single drug, whereas the proportion of polypharmacy (combined use of multiple drugs) in the 12–14 years age group was as high as 17.1%. Oral ingestion was the primary route of poisoning across all age groups. Regarding treatment, the 12–14 years age group had the highest proportion receiving gastric lavage (65.5%), followed by the 1–3 years age group (45.8%). Blood purification was used mainly in the 12–14 years age group, with 89 cases (16.8%), significantly higher than in other age groups. Although children aged 1–3 years constituted the main population of poisoning, the 12–14 years age group accounted for the largest number of hospitalizations and had the highest hospitalization rate (31.8%), compared with only 6.2% in the 1–3 years age group. In the 12–14 years age group, 36 cases (6.7%) were admitted to the pediatric intensive care unit (PICU). Across the entire sample, three in-hospital deaths occurred (one in the 4–6 years age group and two in the 12–14 years age group) (Table 1). Among adolescents aged 12–14 years, poisoning cases are more frequent in September; for the other age groups, the distribution of poisoning cases across months shows no significant difference (Figure 2).

### 3.2. The Evolution of Characteristics of Acute Poisoning from 2019 to 2024

Among 3923 pediatric patients with acute poisoning from 2019 to 2024, the proportion of intentional poisoning increased from 2.94% to 11.83% (z = 5.120, *p* < 0.001), the proportion of psychiatric drug use rose from 0.55% to 15.21% (z = 3.914, *p* < 0.001), the proportion of psychiatric comorbidities increased from 3.31% to 7.85% (z = 2.888, *p* = 0.0039), and the proportion of patients presenting more than 4 h after poisoning increased from 33.82% to 49.30% (z = 9.569, *p* < 0.001). Meanwhile, the proportion of general admissions decreased from 12.87% to 9.05%, and the proportion that received blood purification decreased from 4.04% to 1.49%, both showing significant downward trends (*p* < 0.001). No significant statistical trends were observed for the proportion of female patients or PICU admissions (*p* > 0.05) (Table 2).

#### 3.2.1. Evolution of Acute Poisoning by Age Group

**Figure 3:**
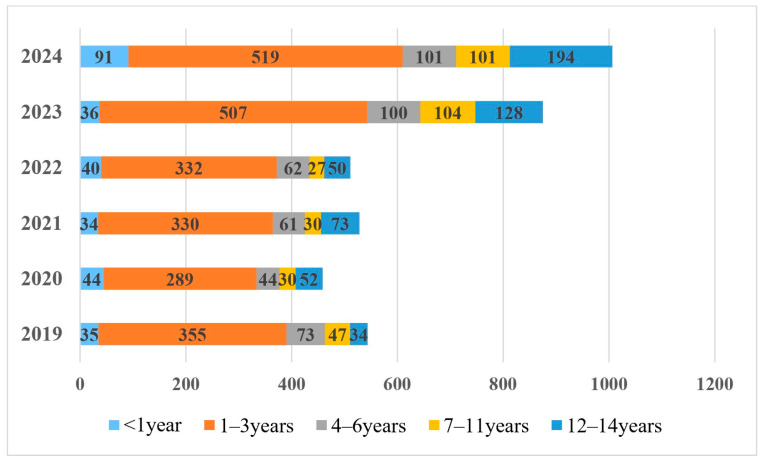
Evolution of acute poisoning by age group.

From 2019 to 2024, the annual number of poisoning cases in each age group gradually increased, with children aged 1–3 years remaining the main affected population. Among them, the number of poisoning cases in the 12–14 years age group increased markedly, from 34 cases in 2019 to 194 cases in 2024 (Figure 3).

#### 3.2.2. Evolution of Intentional Poisoning

**Figure 4:**
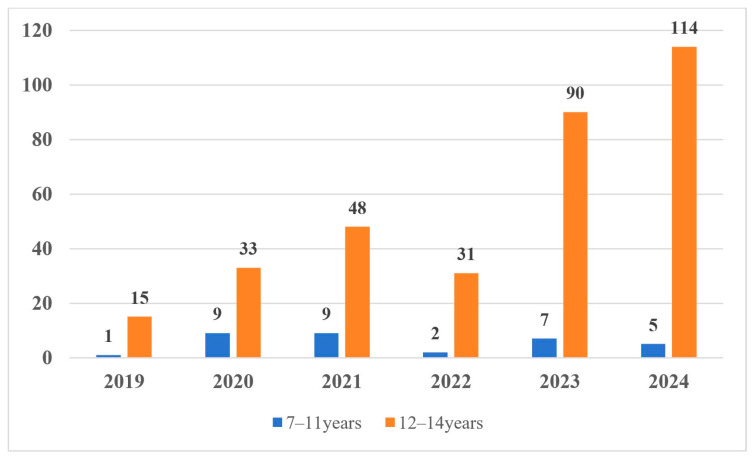
Evolution of intentional poisoning.

In terms of poisoning intent, there were 3559 unintentional poisoning cases and 364 intentional poisoning cases. Among them, intentional poisoning cases were mainly concentrated in the 12–14 years age group. The number of intentional poisoning cases remained relatively stable from 2019 to 2022, but increased significantly in 2023–2024 (Figure 4).

#### 3.2.3. Evolution of the Poison Spectrum

**Figure 5:**
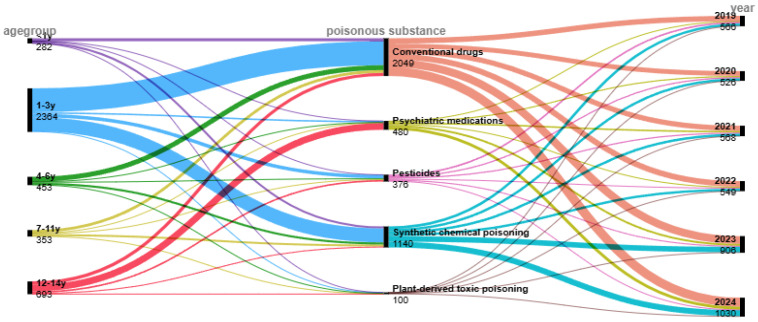
Sankey diagram illustrating the flow and distribution of pediatric acute poisoning cases(The first column represents age groups, with different colors indicating different ages; the second column represents the number of poisoning cases categorized by toxic substance, where a higher column height corresponds to a larger number of cases; the third column represents the year.).

From 2019 to 2024, the poisoning substances in young children remained predominantly household chemicals and non-psychiatric medications. In contrast, psychiatric prescription drugs were the main poisoning substances in the 12–14 years age group, showing an increasing trend from 14 cases in 2019 to 120 cases in 2024 (Figure 5). The classification table of poisons ingested by pediatric patients aged 12–14 years with deliberate poisoning is provided in the Supplementary Materials.

#### 3.2.4. Annual Changes in Psychiatric Comorbidities Among Children Aged 12–14 Years

**Figure 6:**
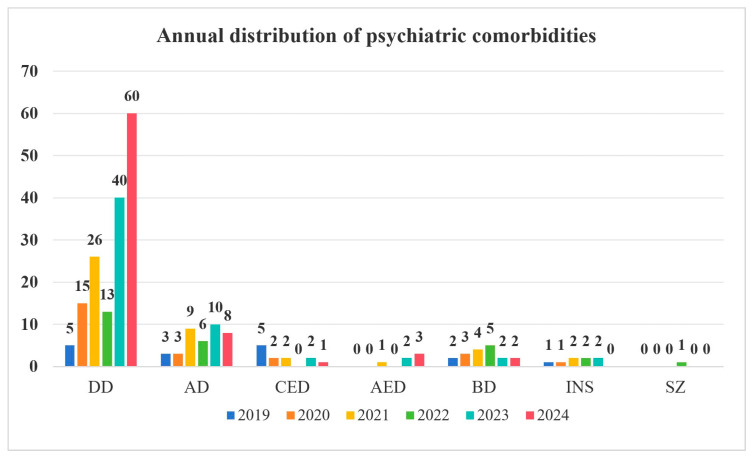
Annual distribution of psychiatric comorbidities among children aged 12–14 years. (DD: Depressive disorder; AD: Anxiety disorder; CED: Childhood emotional disorder; AED: Adolescent emotional disorder; BD: Bipolar disorder; INS: Insomnia; SZ: Schizophrenia).

From 2019 to 2024, the composition and number of psychiatric comorbidities among children aged 12–14 years with intentional poisoning exhibited notable dynamic changes. Regarding the distribution of comorbidity types, depressive disorders (159 cases) and anxiety disorders (39 cases) were the most prevalent in this population, followed by bipolar disorder (18 cases). Conversely, childhood emotional disorders (12 cases), insomnia (8 cases), adolescent emotional disorders (6 cases), and schizophrenia (1 case) accounted for relatively small proportions. In terms of temporal trends, the total number of identified psychiatric comorbidities showed an overall upward trajectory with fluctuations during the study period, with a temporary decline in 2022 before rising sharply again. Notably, depressive disorders demonstrated the most pronounced annual growth, with the number of cases increasing from 5 in 2019 to 60 in 2024 (Figure 6).

#### 3.2.5. Annual Changes in Psychiatric Medications Among Children Aged 12–14 Years

**Figure 7:**
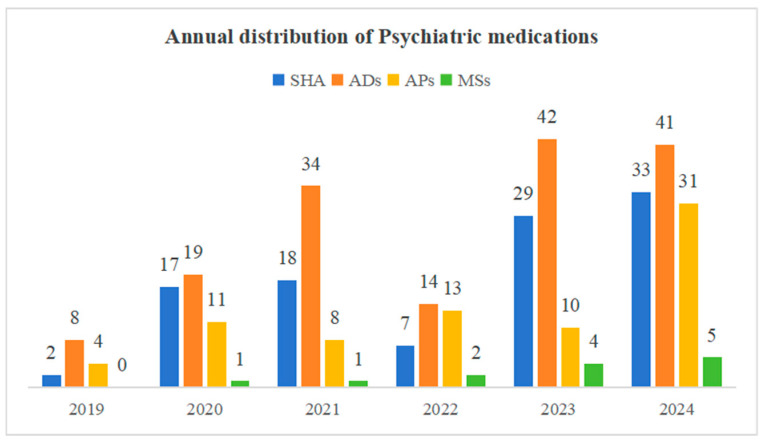
Annual changes in psychiatric medications among children aged 12–14 years (SHA: Sedatives, Hypnotics, and Anxiolytics; ADs: Antidepressants; APs: Antipsychotics; MSs: Mood Stabilizers).

From 2019 to 2024, psychiatric medication-related poisoning cases among children aged 12–14 years increased notably, especially in 2023–2024. Antidepressants (ADs) and sedatives, hypnotics, and anxiolytics (SHA) were the most common agents; antipsychotics (APs) rose sharply in 2024, while mood stabilizers (MSs) remained rare throughout the period (Figure 7).

### 3.3. Analysis of Risk Factors for Intentional Poisoning in the 12–14 Years Age Group

Considering that the risk of intentional poisoning events among children aged 12–14 years was significantly higher than in other age groups, additional stratified analyses were performed focusing specifically on this subgroup.

A total of 531 poisoned children aged 12–14 years were included, among whom 331 (62.3%) engaged in intentional poisoning. The multivariable modified Poisson regression model showed a significant overall fit (Wald χ^2^ = 129.87, df = 7, *p* < 0.001). The results revealed that, after adjusting for other variables, the post-epidemic period (vs. pre-epidemic; RR = 1.55, 95% CI: 1.11–2.19, *p* = 0.011), female gender (vs. male; RR = 1.23, 95% CI: 1.04–1.45, *p* = 0.018), and psychiatric comorbidities (vs. no psychiatric comorbidities; RR = 1.24, 95% CI: 1.08–1.43, *p* = 0.002) were independent risk factors for intentional poisoning. Regarding the types of substances, poisonings by pesticides (RR = 3.67, 95% CI: 2.59–5.19, *p* < 0.001) and psychotropic drugs (RR = 3.27, 95% CI: 2.29–4.66, *p* < 0.001) were associated with a significantly higher risk of intentional behavior compared to other types of drugs. Furthermore, no statistically significant associations were observed for the mid-epidemic period and poisonings by non-steroidal anti-inflammatory drugs (NSAIDs) (all *p* > 0.05) (Table 3).

## 4. Discussion

This study, based on a longitudinal analysis of 3923 cases from 2019 to 2024, reveals a significant “bipolarization” trend in the poisoning spectrum among children. On the one hand, children aged 1–3 years remained the predominant group for poisoning incidents (accounting for 59.4% of cases), characterized by unintentional ingestion in the home environment. On the other hand, a sharp increase in intentional poisoning was observed among adolescents aged 12–14 years during 2023–2024. This polarization reflects differences in risk sources across developmental stages: the toddler group is primarily constrained by physical environmental safety barriers, whereas the adolescent group is profoundly impacted by psychosocial crises [14,15,16,17].

Adolescent mental health is a vital global public health issue with heavy socioeconomic burdens [18,19]. Adolescence is a vulnerable developmental stage for depression onset [20,21], which aligns with our finding that psychiatric comorbidities, especially depression, increased significantly over time among 12–14-year-olds. The most common psychiatric comorbidities are depression and anxiety disorders. Specific psychiatric disorders—such as emotional dysregulation and impulse control disorders—superimpose onto adolescent psychosocial stress, thereby translating into a high risk for deliberate poisoning (self-harm/suicide). The post-pandemic period (2023–2024) was also identified as an independent risk factor for adolescent intentional self-poisoning (ISP). ISP-related emergency visits remained stable during strict pandemic control (2020–2022). After full recovery of social and school operations, however, ISP cases rose sharply, requiring close attention from public health and school mental health systems. Three potential mechanisms account for this trend. Firstly, the COVID-19 pandemic raised public mental health awareness and promoted routine school psychological screening, enabling earlier detection of hidden psychological crises and self-harm after pandemic restrictions were lifted. Secondly, long-term economic uncertainty, family financial pressure and persistent pandemic stress worsened parent–child conflicts and weakened family support, raising adolescents’ risks of emotional dysregulation and self-harm [22,23]. Third, prolonged online learning and overuse of social media reduced adolescents’ offline social and stress-coping abilities. When returning to campus, they faced academic gaps, heavier study loads and intense peer competition, leading to poor adaptation, psychological imbalance and subsequent intentional poisoning [14,15,16,17,22,24,25,26].

Our finding that ISP cases peak every September supports this mechanism, as the new semester brings severe academic and readjustment stress. Academic pressure and school reintegration anxiety are major triggers of ISP, consistent with previous post-pandemic mental health studies [27]. In addition, females had a significantly higher ISP risk than males, and over half of affected adolescents had no prior psychiatric diagnoses. Many young people with subclinical psychological distress are left undetected, and accumulated negative emotions may eventually trigger severe self-harm. These results highlight the necessity of early screening and timely intervention for adolescents’ latent mental health problems.

Another key finding of this study is the evolution of the poisoning spectrum. From 2019 to 2024, the proportion of psychotropic drug poisoning among adolescent ISP cases surged from 0.55% to 15.21%. Psychotropic prescription drugs, represented by fluvoxamine, quetiapine, sertraline, and benzodiazepines, have replaced traditional over-the-counter drugs as the primary poisons in adolescent ISP, with a trend of poly-drug exposure (17.1% of patients involved combined medication). This trend is closely related to the increased diagnosis rate of mental disorders among adolescents, greater accessibility of psychotropic drugs, and inadequate control of home medicine cabinets [28,29]. Complex poison exposure not only complicates clinical management but also reflects a strong intent for deliberate self-harm [28]. The poison spectrum in our adolescent group differs from that reported in foreign studies, where NSAIDs, analgesics and alcohol are the most common, followed by psychotropic drugs [29,30,31]. This discrepancy may be attributed to differences across countries in the regulation of psychotropic drugs, drug accessibility, and preferences for self-harm behaviors among adolescents. Accordingly, it is imperative to strengthen the regulation of psychotropic prescription drugs by standardizing clinical prescribing practices, strictly controlling drug sales channels, enhancing home medication management, improving multi-departmental collaborative supervision, and providing safety training on medication use for patients and caregivers, so as to reduce the risk of deliberate self-harm among adolescents.

Furthermore, our study observed that despite a high volume of intentional poisoning cases in the 12–14 years age group, the number of PICU admissions and deaths remained exceptionally low. This discrepancy is likely because the majority of these intentional poisonings involved the acute ingestion of psychotropic medications, rodenticides, and organophosphate insecticides—toxicants for which relatively effective and reversible supportive treatments are available. Compared to acetaminophen overdoses—which are common in Western countries and can lead to irreversible fulminant hepatic failure if presentation is delayed—the acute lethality of the substances predominantly ingested in our cohort is relatively low. This partly explains why the PICU admission rate and overall mortality did not deteriorate proportionally alongside the surge in poisoning incidents.

Based on the observed evolution of the adolescent intentional poisoning spectrum in this study, the clinical intervention model urgently needs to transition from a single “physical treatment” approach to a comprehensive system integrating “early prevention and psychological intervention” [21]. Next, we discuss the current domestic specifications and clinical practical situations. Developed countries in Europe and America have established and enforced child-resistant packaging specifications through legislation, which systematically reduces the risk of accidental ingestion among children at the legal level [32]. China has issued the national standard GB/T 42089-2022 for child-resistant packaging on non-pharmaceutical products [33], providing technical specifications in this area. While this standard is currently recommended rather than mandatory, China has also actively initiated work on child-safe packaging for chemicals and over-the-counter drugs, with relevant regulations under continuous improvement. These differences in regulatory approaches may partially explain the relatively high incidence of accidental home ingestion among young children in China. Therefore, it is necessary to further strengthen the regulatory system and promote broader implementation of child-safe packaging standards. In addition, clinical observations in this study show that many households store pesticides and daily chemicals in repurposed ordinary beverage bottles without any hazard warning labels. Such irregular home storage practices easily lead to misidentification by young children and subsequent accidental ingestion. Hence, in addition to the standardization of product packaging, it is essential to strengthen safety management and public health education regarding the household storage of daily chemicals and pesticides, so as to reduce the risk of pediatric accidental ingestion in family scenarios.

For older adolescents with underlying mental disorders, caregivers must supervise medication use throughout the treatment process, strictly controlling drug storage and administration to minimize the risk of medication-facilitated self-harm. During high-risk periods, such as the beginning of the academic year in September (when intentional poisoning incidents peak), routine school-based mental health screening and stress management programs should be implemented to promptly identify subclinical psychological crises and facilitate early intervention. Furthermore, emergency department workflows should be optimized to extend beyond acute toxicological management; a mandatory psychological assessment pathway for adolescent poisoning patients must be established. Once intentional poisoning is identified, regardless of the severity of somatic symptoms, timely psychological consultations should be arranged to initiate early intervention, thereby mitigating the risk of subsequent suicide attempts and recurrent poisoning, and ultimately improving long-term prognosis.

This study has several limitations. First, our center primarily admits children aged 14 years or younger; therefore, data on children older than 14 years are not included. Second, this is a single-center retrospective study. The geographical restriction of the case source may introduce selection bias, and the generalizability of the findings is limited. Third, the psychological and behavioral analysis of adolescent poisoning relies solely on medical record documentation, lacking standardized psychological assessment scale data. Future prospective studies incorporating both sociological and psychological tools are needed to explore the underlying triggers. Additionally, because our center is not a specialized psychiatric institution, long-term follow-up data for children and adolescents with intentional poisoning are incomplete, making it difficult to evaluate the recurrence rate and long-term prognosis after intervention. Therefore, multicenter, prospective studies are still required to further validate and refine the conclusions of this study. We noted that there are significant urban-rural disparities in Southwest China. As our hospital is the highest-tier specialized maternal and child health center in the region, it likely receives a higher proportion of complex cases or patients with prominent psychiatric issues, which may introduce a certain degree of referral bias. Additionally, prescription drug regulation policies vary widely across different countries/regions (e.g., opioids, which are common abroad, are extremely rare in our cohort). Therefore, the evolutionary characteristics of the poison spectrum we observed may be most applicable to East Asian countries with similar medical regulatory systems, and extrapolation to other international healthcare systems should be done with caution. This study was a retrospective observational study. The identified risk factors only indicate correlation rather than definite causality. Further prospective cohort studies are required to validate the present findings, and caution is needed when generalizing these results to other populations or settings.

## 5. Conclusions

Most poisoning incidents occur in young children and take place at home. Female adolescents are common patients of intentional poisoning. After the pandemic, the mental health problems of adolescents among pediatric acute poisoning cases have become increasingly prominent. Clinical prevention and control need to shift from simple emergency treatment to early intervention, psychological screening, and comprehensive prevention, so as to reduce health damage to children and the societal disease burden.

## Figures and Tables

**Table 1:** General characteristics.

	Age Group				
	<1 y	1–3 y	4–6 y	7–11 y	12–14 y
**Number of patients**	280	2332	441	339	531
Gender, *n* (%)					
Male	149 (53.2)	1316 (56.4)	233 (52.8)	160 (47.2)	142 (26.2)
Female	131 (46.8)	1016 (43.6)	208 (47.2)	179 (52.8)	389 (73.8)
**Time elapse to ED (Hours), *n* (%)**					
≤4 h	160 (57.1)	1499 (64.3)	211 (47.8)	165 (48.7)	196 (36.2)
>4 h	120 (42.9)	833 (35.7)	230 (52.2)	174 (51.3)	345 (63.8)
**Site of poisoning**					
At home	277 (98.9)	2304 (98.8)	430 (97.5)	318 (93.8)	458 (86.3)
Away from home	0	7 (0.3)	4 (0.9)	10 (2.9)	24 (4.5)
Unknown	3 (1.1)	21 (0.9)	7 (1.6)	11 (3.3)	49 (9.2)
**Comorbidities** **, *n* (%)**					
Psychiatric/psychological	0(0)	0(0)	2(0.5)	18(5.3)	243(45.8)
Others	6 (2.1)	30 (1.3)	4 (0.9)	16 (4.7)	13 (2.4)
**Main type of poisons, *n* (%)**					
Psychiatric drugs	2 (0.7)	82 (3.5)	3 (0.7)	29 (8.6)	364 (67.3)
Common medications	162 (57.9)	1298 (55.7)	265 (60.1)	150 (44.2)	174 (32.2)
Household products	106 (37.9)	760 (32.6)	113 (25.6)	106 (31.3)	55 (10.2)
Pesticides/insecticides	10 (3.6)	189 (8.1)	49 (11.1)	42 (12.4)	86 (15.9)
**Number of substances, *n* (%)**					
Single substance	279 (99.6)	2300 (98.6)	431 (97.7)	332 (97.9)	450 (82.8)
Multiple substances	1 (0.4)	32 (1.4)	10 (2.3)	7 (2.1)	91 (17.1)
**Treatment methods, *n* (%)**					
Gastric lavage	84 (30.0)	1069 (45.8)	149 (33.8)	109 (32.2)	348 (64.3)
Blood purification	0 (0.0)	18 (0.8)	11 (2.5)	13 (3.8)	89 (16.5)
Charcoal	0 (0.0)	89 (3.8)	29 (6.6)	25 (7.4)	102 (18.9)
**Antidote use, *n* (%)**					
Vitamin K1	3 (1.1)	53 (2.3)	22 (5.0)	10 (2.9)	15 (2.8)
Others	0 (0.0)	12 (0.5)	4 (0.9)	11 (3.2)	14 (2.6)
**Intent of poisoning, *n* (%)**					
Accidental poisoning	280 (100)	2332 (100)	441 (100)	306 (90.3)	200 (37.7)
Intentional poisoning	0 (0)	0 (0)	0 (0)	33 (9.7)	331 (62.3)
**Disposition, *n* (%)**					
Ward	9 (3.2)	145 (6.2)	37 (8.4)	51 (15.0)	169 (31.8)
PICU	0 (0.0)	13 (0.6)	3 (0.7)	11 (3.2)	36 (6.7)
Home	271 (96.8)	2174 (93.2)	401 (90.9)	277 (81.1)	326 (61.5)

**Table 2:** Annual Trends of acute poisoning in children (2019–2024).

Year	Sample Size (n)	Intentional Poisoning (%)	Female Patients (%)	Time Elapse to ED > 4 h (%)	Ward (%)	PICU (%)	Blood Purification (%)	Psychiatric Drug (%)	Psychiatric Comorbidity (%)
2019	544	2.94	46.14	33.82	12.87	1.1	4.04	0.55	3.31
2020	459	9.15	48.15	35.73	16.34	2.18	6.75	1.31	5.66
2021	528	10.8	50.57	32.95	18.56	2.46	6.25	1.55	9.28
2022	511	6.46	49.32	33.66	13.5	2.15	3.91	0.99	5.68
2023	875	11.09	48.11	57.37	8.11	0.8	1.14	1.19	7.31
2024	1006	11.83	50.5	49.3	9.05	1.59	1.49	15.21	7.85
Trend z-value	-	5.12	1.243	9.569	−5.064	−0.645	−5.788	3.914	2.888
*p* value	-	<0.001	0.2139	<0.001	<0.001	0.5191	<0.001	<0.001	0.0039

**Table 3:** Analysis of risk factors for deliberate poisoning among children aged 12 to 14 years.

Risk Factor	Reference	RR	95% Confidence Interval	*p*-Value
Mid-epidemic stage	Pre-epidemic stage	1.31	0.93–1.86	0.127
Post-epidemic stage	Pre-epidemic stage	1.55	1.11–2.19	0.011
Female	Male	1.23	1.04–1.45	0.018
Comorbid psychiatric disorders	Without psychiatric comorbidities	1.24	1.08–1.43	0.002
Pesticide poisoning	Other drug poisoning	3.67	2.59–5.19	<0.001
Psychotropic drug poisoning	Other drug poisoning	3.27	2.29–4.66	<0.001
NSAID poisoning	Other drug poisoning	1.56	0.91–2.66	0.106

## Data Availability

Reasonable requests for the datasets used and/or analyzed during the current study should be directed to the corresponding author.

## Supplementary Materials

The following supporting information can be downloaded at: https://www.mdpi.com/article/10.3390/jcm15135309/s1, Table S1: Yearly distribution of toxic substances, 12–14 age group.

## Abbreviations

The following abbreviations are used in this manuscript:

COVID-19	Coronavirus Disease 2019
LR	Likelihood ratio test
RR	Relative risks
95% CIs	95% confidence intervals
PICU	Pediatric intensive care unit
ED	Emergency Department
ISP	Intentional self-poisoning
WHO	World Health Organization
HIS	hospital information system
DD	Depressive disorder
AD	Anxiety disorder
CED:	Childhood emotional disorder
AED	Adolescent emotional disorder
BD	Bipolar disorder
INS	Insomnia
SZ	Schizophrenia
ADs	Antidepressants
SHAs	Sedatives, hypnotics, and anxiolytics
APs	Antipsychotics
MSs	Mood stabilizers
NSAIDs	Non-steroidal anti-inflammatory drugs
EMR	electronic medical record
